# Executive Processes and Emotional and Behavioural Problems in Youths Under Protective Measures

**DOI:** 10.3389/fpsyg.2021.716489

**Published:** 2021-08-19

**Authors:** Juan Manuel Moreno-Manso, M.ª Elena García-Baamonde, Eloísa Guerrero-Barona, M.ª José Godoy-Merino, Mónica Guerrero-Molina, Carlos Barbosa-Torres

**Affiliations:** ^1^Department of Psychology, University of Extremadura, Badajoz, Spain; ^2^Department of Educational Sciences, University of Extremadura, Badajoz, Spain

**Keywords:** executive functions, emotional problems, executive processes, behavioural problems, psychopathology, residential care

## Abstract

This research studies the executive processes of youths under protective measures between 13 and 18years of age, as well as the emotional problems they have and the presence of behavioural problems, such as difficulties to control and direct attention, to control one’s own behaviour and inhibit inadequate or ineffective responses (hyperactivity-impulsiveness) and problems related to emotional regulation. In addition, we study the presence of significant differences according to the sex of the youths. We also analyse to what extent the difficulties in the executive processes are related to and can predict the emotional and behavioural problems. The instruments used were Stroop’s Colour and Word Test (Stroop), the Paths Test (TESen), and the System of Evaluation for Children and Adolescents (SENA). The results indicated that the youths had difficulties in such executive processes as execution, speed, and accuracy in carrying out tasks. Furthermore, they had emotion problems, amongst which the symptoms of anxiety are worthy of note; whilst attention deficit, hyperactivity-impulsiveness, and problems related to emotional regulation could also be observed. The data indicated greater difficulties in the executive processes for males than for females. There was a greater emotional symptomatology in the females, whilst there were greater deficits in attention and hyperactivity/impulsiveness in the males. Similarly, the deficits in the executive processes were related to and predicted emotional and behavioural problems. This research suggests the design of a structured programme focused on systematic training in real, daily situations, recommending the use of restorative techniques to work on the affected cognitive skills and techniques aimed at improving the youths’ emotion regulation.

## Introduction

Residential care is a protective measure aimed at those youths who, due to the especially vulnerable situation, cannot remain in the family homes. These youths are in a situation of abuse or serious risk of suffering abuse, either because the families do not or cannot adequately attend to the basic needs, or because they are victims of abuse. It is thus necessary to provide them with a temporary place of residence that can guarantee them the necessary protection, education and emotional, cognitive, behavioural, and social development.

Many of these youths may present negative psychological consequences because of their situation of abuse and/or their adverse family life (D’Andrea et al., 2012; Jennissen et al., 2016; Jozefiak et al., 2016). Green et al. (2010), Cicchetti and Toth (2016), and Jaffee (2017) point out that child abuse explains the risk of suffering a psychopathology in a very high percentage of cases. Teicher and Samson (2013), Hodgdon et al. (2018), Karatekin et al. (2018), and Racine et al. (2020) point out that the victims of abuse have greater possibilities of developing such psychological symptoms and disorders as depression, anxiety, post-traumatic stress, psychosomatic disorders, attention deficit hyperactivity disorder (ADHD), behavioural disorders, personality disorders, psychosis, substance abuse, or suicidal tendencies, amongst others. Sege et al. (2017) and Docherty et al. (2018) point to the most frequent emotional and behavioural manifestations in victims of abuse as being attention deficits, oppositional defiant disorders, an inability to regulate emotional states, problems with the peers, and unruly behaviour at school.

Psychosocial adversities suffered at an early age such as the child abuse, may be prejudicial for the neurobiological systems, perturbing the development of the neuronal circuits and interfering in the brain’s development (De Bellis et al., 2013; Rock et al., 2018), thus increasing one’s vulnerability to mental health problems (Kim and Cicchetti, 2010; De Bellis and Zisk, 2014; Greger et al., 2015). Similarly, it has been observed that youths with psychopathology symptomatology, caused by situations of psychosocial stress, present structural alterations in certain regions of the brain such as the amygdala, the prefrontal cortex, and the hippocampus (Grant et al., 2014; Kavanaugh et al., 2017; Quinlan et al., 2017). Carrión et al. (2010) and Tottenham et al. (2011) showed that youths who have suffered traumatic experiences present a lower activity in the prefrontal cortex. These works concluded that there were neuronal losses in the medial prefrontal cortex, a region involved in the executive functions. Davis et al. (2015) and Cromer and Villodas (2017) demonstrated that alterations in the prefrontal regions occasioned by such traumatic experiences as abuse have emotional and behavioural consequences for the victims.

Concerning the differences between the genders, one of the limitations of studies is the use of samples of only one gender. However, some studies have found a greater effect in females related to cognition and emotional regulation; whilst in males, worse control over one’s impulses has been found (Edmiston et al., 2011; Tung et al., 2012). Camuñas et al. (2020) observed more emotional problems in females than in males. Teicher et al. (2004), Shea et al. (2005), and Burghy et al. (2012) found a greater probability of internalising problems (anxiety, depression, post-traumatic stress…) in females and of externalising problems in males (attention deficits and hyperactivity).

In this context, the objectives of the research were: to analyse the executive processes of youths under protective measures, together with the emotional problems they may present, and the presence of behavioural problems, such as difficulties in controlling and directing attention, in controlling one’s own behaviour and inhibiting inadequate or ineffective responses (hyperactivity-impulsiveness) and problems related to emotional regulation; to verify the presence of significant differences due to gender in the youths’ executive processes, emotional and behavioural problems; to analyse the relationship between the executive processes, emotional difficulties, and the behavioural problems; and to determine the extent to which the executive processes can predict emotional problems and the behavioural problems. Based on the theoretical review carried out, we expected the youths to show deficits in the executive processes, emotional problems, and the behavioural problems (hypothesis 1). Furthermore, we expected significant differences in the executive processes, emotional problems, and the behavioural problems according to gender (hypothesis 2). We also expected difficulties in the executive processes to be related to the emotional problems and the behavioural problems (hypothesis 3). Finally, we expected the deficits in the executive processes would be able to act as predictors of the emotional difficulties and the behavioural problems (hypothesis 4).

## Materials and Methods

### Participants

The sample consisted of 61 youths between 13 and 18years of age (*Mean*=14.98; *SD*=1.21) in residential care with protective measures. About 47.5% of the participants were female (*n*=29) and 52.5% were male (*n*=32). The participants made up the total number of youths within this age range in public residential care and in privately managed tutored flats in the Region of Extremadura (Spain) in 2019. The sample size was adequate, given that the number of participants is representative of the population in residential care in Spain (Observatorio de la Infancia, 2019). The average period of time spent in residential care centres was 38months.

The reason for the protective measures and the corresponding admittance to a residential care centre was the legal situation of vulnerability. A total of 33 youths (54.1%) had been separated from the family due to child abuse. As for the type of abuse, 21.5% of the participants had suffered physical neglect, followed by physical and emotional neglect (14.3%), physical abuse (9.8%), physical and emotional abuse (7%), and sexual abuse (1.5%). In the remaining 45.9% of youths (*n*=28), the protective measure was for other reasons, such as the impossibility of the parents or legal tutors to carry out the obligations towards them (alcoholism, drug consumption, prison, mental illness, prostitution, or domestic violence; 25.2%), the abandonment/express renunciation on the part of the parents/tutors (3%) or due to an inability on the parents’ part to control the youth’s behaviour (17.7%).

As for the youths’ educational situation, it should be pointed out that 73.7% were in obligatory secondary education, 3.3% were in baccalaureate, 9.7% were in vocational training, and 13.3% were not studying at all.

No youths receiving therapeutic attention following a diagnosis of neurodevelopmental disorders, such as intellectual disability, disorders of the autistic spectrum, ADHD, or specific learning disorders, took part in the research, thus avoiding the bias that the inclusion would have supposed for the objectives of the study.

The information concerning diagnoses of the youths’ physical or mental health was obtained from the personal files of the Social Services of the Region of Extremadura (Spain). The youths who participated in the research presented no relevant physical or mental health problems and no mention was made in the files of the presence of intellectual disability.

### Instruments

#### Colour and Word Test (Stroop)

This is one of the most widely used tests to detect neuropsychological problems or brain damage and to evaluate interference (Golden, 2007). The test consists of three tasks: reading words, naming colours, and a final task of interference. A comparison of the scores obtained in the three tasks allows the effects of interference in the subjects and the attention control to be evaluated. The test involves the ability to select relevant information in a flexible way and adapt to new circumstances, to face cognitive stress and process complex information, inhibitory control, the speed of processing, the ability to plan and execute strategies aimed at achieving a goal, working memory, and cognitive flexibility. The test is applied individually and is sensitive to lesions in the frontal lobe. The tasks involve focusing on a particular stimulus (selective attention) and ignoring distractions (irrelevant stimuli; inhibitory control). As for the normative data, the average scores are between 40 and 60. With respect to the reliability data, Cronbach’s Alpha in the three sheets is, respectively, *α*=0.85, *α*=0.81, and *α*=0.69; whilst in interference it is *α*=0.70.

#### The Paths Test (TESen)

The Paths Test (TESen; Portellano and Martínez, 2014) is a screening test aimed at detecting alterations in the executive functioning. The instrument consists of carrying out a planning task of hand-eye coordination. The test is applied individually and consists of four parts (paths) of increasing difficulty. The tasks that must be carried out in each part involve, progressively, attention and executive processes of varying complexity. The test provides us with scores concerning speed (the ability to resolve tasks that require increasing amounts of attention and executive control), accuracy (successes and errors committed), and execution (efficiency with which the task is carried out). As for the normative data, the average scores are between 40 and 60; whilst for the reliability data, Cronbach’s Alpha in speed is *α*=0.93, in accuracy *α*=0.89 and in execution *α*=0.88.

#### System of Evaluation for Children and Adolescents

This instrument provides very useful information for detecting psychological problems in children and adolescents (Fernández-Pinto et al., 2015). The test is aimed at evaluating emotional and behavioural problems, as well as contextual problems. However, considering the aims of our study, contextual problems were not evaluated. With respect to emotional problems, it evaluates depression, anxiety, social anxiety, somatic problems, post-traumatic symptomatology, and obsession-compulsion. As for the behavioural problems, it evaluates problems of attention, hyperactivity-impulsiveness, and emotion control. With respect to the normative data of this instrument, the average is 50 and the SD is 10. The instrument’s reliability reflects adequate indices. The mean of Cronbach’s Alpha is between *α*=0.82 and *α*=0.85 on the scales of samples from the general and clinical populations, respectively, and of *α*=0.93 in the global indices.

### Procedure

The research was authorised by the institution responsible for the legal tutelage of the youths (Region of Extremadura, Spain). All procedures performed were in accordance with the ethical standards of Extremadura University (Ref.: 181/2020) and with the 1964 Helsinki declaration and its later amendments or comparable ethical standards. All subjects gave the informed consent for inclusion before they participated in the study.

The evaluators received prior formation on applying the instruments so as to guarantee the validity and reliability of the data gathered. All the instruments were applied individually in the centres and homes where the youths resided. No difficulties arose, whilst the tests were being carried out.

### Statistical Analyses

This is a descriptive study of a transversal nature. We first of all carried out a descriptive analysis of the youths’ executive processes, the emotional problems, and the behavioural problems. Secondly, having confirmed the use of non-parametric tests, we then used the Mann-Whitney U test to analyse the presence of significant differences according to gender. Thirdly, we performed a Spearman’s correlation analysis to analyse the relation between the executive processes, the emotional problems, and the behavioural problems; finally, we carried out a linear regression analysis to determine the extent to which the executive processes can predict emotional difficulties and behavioural problems.

The statistical package SPSS version 25 was used for the statistical treatment of the data.

## Results

Table 1 shows the descriptive data from the Stroop, TESen, and System of Evaluation for Children and Adolescents (SENA).

**Table 1:** Means and SDs of the Stroop, TESen, and System of Evaluation for Children and Adolescents (SENA).

	*Mean*	*SD*
**Stroop**		
Words	40.92	8.14
Colours	36.02	7.65
Words-colours	38.93	5.90
Interference	45.80	2.79
TESen		
Execution	29.51	4.35
Speed	27.87	5.28
Accuracy	24.67	4.06
**SENA**		
Global indices		
Index of emotional problems	63.62	2.72
Index of behavioural problems	64.48	2.07
**Emotional problems**		
Depression	52.31	4.29
Anxiety	63.90	2.56
Social anxiety	60.77	2.62
Somatic problems	61.77	5.29
Post-traumatic symptomatology	60.41	8.81
Obsessive–compulsive	58.16	7.15
**Behavioural problems**		
Attention problems	64.38	4.84
Hyperactivity-impulsiveness	64.46	6.58
Problems controlling emotions	63.08	5.81

The data from Stroop test showed mean scores of below 50, which is indicative of a low resistance to interference and thus less inhibitory attention control. Several of the youths show difficulties to inhibit or control automatic responses. The low scores observed may indicate scarce cognitive flexibility and a low adaptation to cognitive stress required in new situations, as well as a lower capacity to take decisions and resolve problems.

Results from TESen test indicated that the global performance in the test is below of the normative group in execution, speed, and accuracy. The scores in speed were below the normal range, indicating that there are significant differences in the speed of cognitive processing, in cognitive, attention, visuospatial, and psychomotor fluency. The scores in accuracy were the lowest. This indicates difficulties in planning and solving more difficult problems and is caused by insufficient mental flexibility (rigidity), inhibitory deficit (impulsiveness or low resistance to interference), or difficulties in working memory. This may be related to a deficient control of emotional regulation and impulses.

Results from SENA test indicated that the global indices the scores obtained were above average in both indices (emotional problems and behavioural problems). The highest score is in the index of behavioural problems, which is one of the most severe difficulties.

The data indicated scores higher than the normative group in such emotional problems as anxiety, social anxiety, somatic problems, and post-traumatic symptomatology. It is also worth mentioning the high scores in problems of attention, hyperactivity-impulsiveness, and problems with regulating emotions.

As for gender differences in the executive processes, in emotional problems and in behavioural problems, Table 2 shows the data from the Mann-Whitney U test.

**Table 2:** Mann-Whitney U test relating to the executive processes and emotional and behavioural problems according to gender.

	Male		Female			
	*Mean*	*SD*	*Mean*	*SD*	*Z*	*p*
**Stroop**						
Words	39.47	8.77	42.52	7.19	−1.27	0.203
Colours	35.66	8.53	36.41	6.67	−0.65	0.510
Words-colours	37.25	4.85	40.79	6.46	−2.03	0.042
Interference	44.44	2.46	47.31	2.36	−4.03	0.000
**TESen**						
Execution	28.28	3.93	30.86	4.44	−2.41	0.016
Speed	25.31	4.90	30.69	4.16	−3.97	0.000
Accuracy	22.34	3.58	27.24	2.86	−4.88	0.000
SENA						
**Global indices**						
Index of emotional problems	61.59	1.84	65.86	1.50	−6.19	0.000
Index of behavioural problems	66.31	0.69	62.45	0.78	−7.04	0.000
**Emotional problems**						
Depression	51.47	4.00	53.24	4.48	−1.16	0.246
Anxiety	62.13	1.96	65.86	1.48	−5.81	0.000
Social anxiety	59.59	2.46	62.07	2.17	−3.66	0.000
Somatic problems	60.97	6.19	62.66	3.99	−1.44	0.149
Post-traumatic symptomatology	58.00	8.60	63.07	8.40	−4.35	0.000
Obsessive-compulsive	56.44	6.70	60.07	7.27	−1.91	0.056
**Behavioural problems**						
Attention problems	66.38	2.49	62.17	5.81	−3.65	0.000
Hyperactivity-impulsiveness	67.41	4.44	61.21	7.07	−3.40	0.001
Problems controlling emotions	60.69	6.87	65.72	2.50	−3.60	0.000

Results from Stroop test showed significant differences according to gender in words-colours and interference. The average score in the males was lower than that of the females.

The data from TESen test indicated significant differences according to gender in executive processes analysed (execution, speed, and accuracy). The scores were lower for the males than for the females. The males thus presented greater difficulties than the females.

Results from SENA test in the global indices showed significant differences in the index of emotional problems and in the index of behavioural problems. The females had more emotional difficulties than the males, whilst the males had more behavioural problems than the females.

Significant differences were observed in anxiety, social anxiety, and post-traumatic symptomatology. The females showed greater emotional symptomatology.

The results showed significant differences in problems of attention and hyperactivity-impulsiveness. The males presented a higher externalising symptomatology than the females. As for emotional problems, the females had more problems here than the males.

With respect to the relation between the executive processes, emotional problems, and the behavioural problems, Table 3 shows the correlation data.

**Table 3:** Correlation analysis between the executive processes, emotional problems, and behavioural problems.

	Stroop				TESen		
	Words	Colours	Words-colours	Interference	Execution	Speed	Accuracy
**Global indices**							
EMO	0.227	0.050	−0.297^*^	−0.467^**^	0.158	−0.350^**^	−0.386^**^
BEH	−0.161	−0.238	−0.238	−0.416^**^	−0.387^**^	−0.581^**^	−0.660^**^
**Emotional problems**							
DEP	0.062	−0.047	−0.044	0.084	0.039	0.121	0.053
ANS	0.208	0.052	0.229	−0.376^**^	−0.321^*^	−0.463^**^	−0.494^**^
ASC	0.105	0.070	0.164	0.201	0.112	−0.288^*^	0.183
SOM	0.085	−0.027	0.166	0.168	0.031	0.121	−0.011
PST	0.124	0.155	−0.268^*^	−0.254^*^	0.117	−0.307^*^	−0.322^*^
OBS	0.090	0.221	0.211	0.231	0.125	0.089	0.194
**Behavioural problems**							
ATE	0.097	0.209	−0.103	−0.485^**^	−0.281^*^	0.165	0.031
HIP	0.106	0.043	−0.034	−0.250	−0.266^*^	0.127	0.008
REG	0.117	0.092	0.142	−0.408^**^	0.184	−0.432^**^	−0.419^**^

*EMO: Index of emotional problems; BEH: Index of behavioural problems; DEP: Depression; ANS: Anxiety; ASC: Social anxiety; SOM: Somatic problems; PST: Post-traumatic symptomatology; OBS: Obsessive-Compulsive; ATE: Attention problems; HIP: Hyperactivity-impulsiveness; REG: Problems controlling emotions*.

* *p*<0.05;

** *p*<0.01.

The results indicated a significant negative correlation between the index of emotional problems and the executive processes, such as those for words-colours (*p*=0.020), interference (*p*=0.000), speed (*p*=0.006), and accuracy (*p*=0.002). Anxiety correlates with the executive processes of interference (*p*=0.003), execution (*p*=0.012), speed (*p*=0.000), and accuracy (*p*=0.000); social anxiety correlates with speed (*p*=0.024); and post-traumatic symptomatology correlates with words-colours (*p*=0.036), interference (*p*=0.048), speed (*p*=0.016), and accuracy (*p*=0.011).

With respect to the index of behavioural problems, the data indicated a significant negative correlation with interference (*p*=0.001), execution (*p*=0.002), speed (*p*=0.000), and accuracy (*p*=0.000). Attention correlates with the processes of interference (*p*=0.000) and execution (*p*=0.028); hyperactivity-impulsiveness correlates with execution (*p*=0.038); and emotional problems correlates with interference (*p*=0.001), speed (*p*=0.001), and accuracy (*p*=0.001).

Finally, we carried out a linear regression to identify whether the deficits in the executive processes can act as predictors of emotional problems and the behavioural problems (Table 4).

**Table 4:** Regression analysis concerning the executive processes and emotional problems and behavioural problems.

	EMO				BEH			
	R^2^	β	*t*	*Sig*.	R^2^	β	*t*	*Sig*.
**Stroop**								
Words	0.05	0.22	1.77	0.081	0.04	−0.21	−1.69	0.096
Colours	0.00	0.01	0.10	0.916	0.03	−0.17	−1.36	0.179
Words-colours	0.10	−0.31	−2.55	0.013	0.08	−0.28	−2.27	0.027
Interference	0.20	−0.45	−3.95	0.000	0.19	−0.43	−3.72	0.000
**TESen**								
Execution	0.02	0.16	1.24	0.219	0.13	−0.36	−2.97	0.004
Speed	0.12	−0.35	−2.90	0.005	0.33	−0.57	−5.38	0.000
Accuracy	0.13	−0.37	−3.07	0.003	0.41	−0.64	−6.42	0.000

*EMO: Index of emotional problems; BEH: Index of behavioural problems*.

The results showed that, in the index of emotional problems, the processes involved in the tasks of words-colours (*β*=−0.31; *p*=0.013), interference (*β*=−0.45; *p*=0.000), speed (*β*=−0.35; *p*=0.005), and accuracy (*β*=−0.37; *p*=0.003) act as predictors.

It can also be seen that, in the index of behavioural problems, the processes involved in the tasks of words-colours (*β*=−0.28; *p*=0.027), interference (*β*=−0.43; p=0.000), execution (*β*=−0.36; *p*=0.004), speed (*β*=−0.57; *p*=0.000), and accuracy (*β*=−0.64; *p*=0.000) act as predictors.

Similarly, in emotional problems, we found that interference (*β*=−0.35; *p*=0.005), execution (*β*=−0.33; *p*=0.009), speed (*β*=−0.46; *p*=0.000), and accuracy (*β*=−0.46; *p*=0.000) all act as predictors of anxiety; whilst the tasks of words-colours (*β*=−0.27; *p*=0.035), interference (*β*=−0.28; *p*=0.028), and speed (*β*=−0.34; *p*=0.007) act as predictors of social anxiety.

As for behavioural problems, we found that the tasks of colours (*β*=−0.27; *p*=0.031), interference (*β*=−0.51; *p*=0.000), and execution (*β*=−0.33; *p*=0.008) all act as predictors of problems with attention; whilst interference (*β*=−0.36; *p*=0.004) and execution (*β*=−0.26; *p*=0.042) act as predictors of hyperactivity-impulsiveness.

Finally, interference (*β*=−0.37; *p*=0.003), speed (*β*=−0.37; *p*=0.003), and accuracy (*β*=−0.35; *p*=0.005) act as predictors of problems with emotional control.

## Discussion

The objectives of the present research were to analyse the executive processes of youths under protective measures, together with the emotional and behavioural problems, to study the presence of significant differences due to the youths’ gender, and to analyse the extent to which the difficulties in the executive processes are related to and can predict the emotional problems and the behavioural problems.

Based on the results, the youths under protective measures had difficulties in the executive processes, resulting in emotional problems and behavioural problems.

The youths had difficulties in different specific executive processes. The performance was low in the tasks that involve execution, speed, and accuracy, and a low resistance to interference could also be observed. Thus, the youths had problems with processing information, attention control, planning behaviour, working memory, and inhibiting one’s behaviour. In addition, they had scarce cognitive flexibility or adaptation to cognitive stress required in new situations, as well as a lower capacity for taking decisions, resolving problems, and looking for alternatives. In general, these difficulties were greater in males than in females. In this sense, Davis et al. (2015) also found problems in behaviour planning and decision-taking in abused youths; whilst Spann et al. (2012), Nikulina and Widom (2013), and Mothes et al. (2015) showed evidence of problems with mental flexibility, difficulties in problem solving and a low resistance to interference.

With respect to the emotional problems and the behavioural problems, the research demonstrated the presence of symptoms of anxiety, social anxiety, somatic problems, post-traumatic symptomatology, hyperactivity-impulsiveness, attention deficit, and problems regulating the emotions. Furthermore, there was a greater prevalence in females for emotional problems and for regulating emotional control than in males. However, in the case of males, there was a greater prevalence of problems in attention control and hyperactive and impulsive behaviour patterns than in females. The studies carried out by Greger et al. (2015), González-García et al. (2017), and Martín et al. (2020) also found emotional and behavioural problems in youths in residential care.

The youths presented anxiety symptomatology characterised by nervousness and recurrent worries. The research also demonstrated the presence of anxiety symptoms related to situations of a social nature, in both interactions with peers and when they participate in other social situations. In addition, there was evidence of somatic signs and the presence of post-traumatic symptomatology in some youths. The low resistance to interference may be related to the problems of anxiety, given the difficulties the youths had in resolving internal and interpersonal problems and the tendency to see themselves as having more problems than other people. Cougle et al. (2010) and Gardner et al. (2019) found a greater risk of youths suffering from abuse presenting anxiety symptoms or disorders. Bruce et al. (2011) found a relation between adverse experiences in childhood and the presence of social anxiety symptoms, whilst Kealy et al. (2018) found similar results concerning somatic problems.

As for post-traumatic stress disorder, different studies present it as one of the commonest consequences of stressful psychosocial situations (Rock et al., 2018), relating it in turn to alterations in the regulation of one’s emotions (Barlow et al., 2017). As pointed out by these studies, these youths would have a worse performance in areas related to cognitive functioning, such as the executive functions.

With respect to the behavioural problems, the research concluded that the youths showed symptoms of inattention characteristic of ADHD, related to the difficulty of directing one’s attention to tasks and inhibiting the interference of irrelevant stimuli, as well as the difficulty to maintain one’s attention over prolonged periods of time. Hyperactive and impulsive behaviour patterns common in ADHD stand out. Several youths presented an excessive level of motor activity (hyperactivity), accompanied by difficulties with inhibiting one’s behaviour (impulsiveness). The low resistance to interference could be indicative of the difficulties the youths have to inhibit or control the automatic responses; it could also be related to the results in hyperactivity-impulsiveness. They had a greater tendency to develop rigid and defensive behaviour patterns. Becker-Blease and Kering (2016) considered that some of the most frequent consequences of exposition to stressful psychosocial situations in childhood are attention deficits, hyperactivity, and impulsiveness. However, Stern et al. (2018) stated that victimisation may be associated with the diagnosis of ADHD in youths.

Concerning emotional regulation, the youths showed difficulties to identify, understand, and regulate the own emotions, as well as frequent, brusque changes in mood and ups and downs throughout the day. Beers and De Bellis (2002), Cicognani (2011), Wilson et al. (2011), and DeGregorio (2012) also found problems to regulate emotions and impulsiveness.

The research also demonstrated the presence of greater difficulties for males than for females in the executive processes. In females, there was a greater emotional symptomatology (anxiety, social anxiety, and post-traumatic stress); whilst males had greater externalising difficulties, such as attention deficits and hyperactivity/impulsiveness. Teicher et al. (2003) and De Bellis (2005) also observed a different vulnerability in females and males suffering abuse when they have to face stressful experiences in life.

We have found a significant relation between the problems that the youths have in the executive processes, the emotional problems, and the behavioural problems. In the youths with low scores in accuracy, execution, speed, and interference, there exists a greater presence of emotional symptomatology (anxiety, social anxiety, and post-traumatic stress) and behavioural problems (problems of attention and hyperactivity-impulsiveness). The research also demonstrated that the deficits in the executive processes act as predictors of the emotional problems and of the behavioural problems.

The research also found the repercussions of the youths in the executive processes necessary for decision-taking, establishing goals, planning behaviour, adapting to new situations, and regulating one’s emotions. In this sense, Spann et al. (2012) and Heleniak et al. (2016) pointed out that the difficulties in the processes responsible for directing, guiding, and controlling the cognitive, emotional, and behavioural functions could be explained by alterations in the brain’s prefrontal regions, which are responsible for the executive functions.

Regarding the limitations of the research, we must point out that, as the study is transversal, it has not been possible to consider the evolution of the problems, the youths suffered from the moment of the admittance to residential care. No psychological evaluation was carried out upon the admittance, so it has not been possible to establish whether the symptomatology they presented at that moment was a clear result of the situation of abuse, or whether it was a consequence of the time spent in residential care, or of other factors that may have intervened because of the separation from the nuclear family. It has not been possible to determine whether some of the youths had cerebral lesions prior to entering residential care, given that the files do not provide this information. We must also point out, as a limitation, the lack of a control group with which to compare the results. The results have not considered either the type of child abuse or the protective measures when examining differences in the groups. Furthermore, given the size of the sample, we do not consider it opportune to set limits with respect to the type of abuse and/or the protective measures. It would seem pertinent to do so in future research.

With respect to the instruments used to evaluate the youths’ executive processes, we should point out that, in the research, we did consider the appropriateness of the tests, given the socio-cultural characteristics of the participants. Similarly, the tests were applied in the youths’ residential centres/homes, as places of reference, thus avoiding any possible contextual influence that might affect the results.

This research has contributed to the identification of the emotional difficulties and the behavioural problems of the youths under protective measures. Based on the results found, we shall be able to design concrete interventions in the affected areas. To do so, the participation of the educators will be vital, as they are persons of reference for the youths in the residential care centres. Similarly, to carry out the intervention, the design of a structured programme from an ecological and functional perspective, focused on the systematic training of real activities, is essential (Whittaker et al., 2016). It will also be fundamental to work on awareness raising of the deficit and an adjusted perspective of the reality (James, 2011; Rath et al., 2011).

In this sense, it will be necessary to respond to the problems the youths have in the executive processes. We would recommend the use of restoration techniques, since the degree of deterioration is not too severe and the affected cognitive skills can be retrained, through training in real situations, thus providing them with strategies that will allow them to regulate the behaviour, whilst carrying out daily activities. In this way, the youths will have tools for resolving the conflicts that arise in the daily lives, enabling them to identify the problem, regulate the behaviour and look for alternatives before acting. Thus, they will be able to use the internal language (reflecting on the consequences of the behaviour and thus avoiding impulsive decisions).

In conclusion, we can say that the youths under protective measures had difficulties in the executive processes, emotional difficulties and behavioural problems, which may have serious implications for the personal and social development. Davis et al. (2015), Hanson et al. (2015), and Vasilevski and Tucker (2016) also concluded that victimisation may have both short- and long-term consequences for the emotional, social, cognitive, and behavioural development. However, certain circumstances such as the age at which abuse starts, its duration and persistence over time, the attachment to the abuser, as well as the separation from the nuclear family and the admittance to residential care, may cause the presence or not of psychopathology (Spratt et al., 2012).

## Data Availability Statement

The raw data supporting the conclusions of this article will be made available by the authors, without undue reservation.

## Ethics Statement

The studies involving human participants were reviewed and approved by University of Extremadura Ref.: 181/2020. Written informed consent to participate in this study was provided by the participants’ legal guardian/next of kin.

## Author Contributions

JM-M, MG-B, EG-B, and MGo-M: conceptualization and methodology. JM-M, MG-B, CB-T, and MGu-M: data curation. JM-M, CB-T, and MGu-M: formal analysis. JM-M, MG-B, and EG-B: supervision. JM-M, MG-B, and CB-T: writing – original draft preparation. JM-M, MG-B, EG-B, MGo-M, CB-T, and MGu-M: writing – review and editing. All authors contributed to the article and approved the submitted version.

## Conflict of Interest

The authors declare that the research was conducted in the absence of any commercial or financial relationships that could be construed as a potential conflict of interest.

## Publisher’s Note

All claims expressed in this article are solely those of the authors and do not necessarily represent those of their affiliated organizations, or those of the publisher, the editors and the reviewers. Any product that may be evaluated in this article, or claim that may be made by its manufacturer, is not guaranteed or endorsed by the publisher.
